# Dynamic reprogramming of chromatin accessibility during *Drosophila *embryo development

**DOI:** 10.1186/gb-2011-12-5-r43

**Published:** 2011-05-11

**Authors:** Sean Thomas, Xiao-Yong Li, Peter J Sabo, Richard Sandstrom, Robert E Thurman, Theresa K Canfield, Erika Giste, William Fisher, Ann Hammonds, Susan E Celniker, Mark D Biggin, John A Stamatoyannopoulos

**Affiliations:** 1 Department of Genome Sciences, University of Washington, Foege S310A, 1705 NE Pacific Street, Box 355065, Seattle, WA 98195, USA; 2 Genomics and Life Sciences Divisions, Lawrence Berkeley National Laboratory, One Cyclotron Road, MS 84-171, Berkeley, California 94720, USA

## Abstract

**Background:**

The development of complex organisms is believed to involve progressive restrictions in cellular fate. Understanding the scope and features of chromatin dynamics during embryogenesis, and identifying regulatory elements important for directing developmental processes remain key goals of developmental biology.

**Results:**

We used *in vivo *DNaseI sensitivity to map the locations of regulatory elements, and explore the changing chromatin landscape during the first 11 hours of *Drosophila *embryonic development. We identified thousands of conserved, developmentally dynamic, distal DNaseI hypersensitive sites associated with spatial and temporal expression patterning of linked genes and with large regions of chromatin plasticity. We observed a nearly uniform balance between developmentally up- and down-regulated DNaseI hypersensitive sites. Analysis of promoter chromatin architecture revealed a novel role for classical core promoter sequence elements in directing temporally regulated chromatin remodeling. Another unexpected feature of the chromatin landscape was the presence of localized accessibility over many protein-coding regions, subsets of which were developmentally regulated or associated with the transcription of genes with prominent maternal RNA contributions in the blastoderm.

**Conclusions:**

Our results provide a global view of the rich and dynamic chromatin landscape of early animal development, as well as novel insights into the organization of developmentally regulated chromatin features.

## Background

The progressive restriction of cellular fate is a hallmark of development and is believed to involve the sequential modification and perpetuation of chromatin states [1]. However, it is currently unclear how this process unfolds at the level of chromatin structure, and whether early development is characterized chiefly by temporal restriction of a large potential pool of accessible chromatin elements or the progressive acquisition of potential manifested in the timed appearance of novel elements, or a combination thereof.

The *Drosophila melanogaster *embryo is one of the best characterized systems for addressing this challenge. During the first 11 hours of development, a single diploid cell, the fertilized egg (0 hours) undergoes nuclear division to form a blastoderm of approximately 6,000 undifferentiated cells (3 to 4 hours), followed by further division and differentiation into 40,000 cells organized into specific tissues such as nerve, muscle and epithelia (11 hours) [2,3]. This morphological patterning is directed by a temporally ordered regulatory cascade [4-8]. Initiated by a few maternally supplied regulatory proteins, by the blastoderm stage some 40 or so sequence-specific transcription factors control the spatial and temporal expression of around a thousand genes [9-14]. By 11 hours, several hundred regulatory factors, many expressed in narrow subsets of cells, direct transcription of approximately 8,000 genes in patterns so intricate that they often change even between adjacent cells of the same cell type. An additional cohort of several hundred ubiquitously expressed transcription factors act throughout embryogenesis to facilitate the action of stage-selective regulators at promoters, enhancers, insulators and other *cis*-acting elements.

To understand the developmental control of transcription and morphogenesis, it is critical to identify the full set of sequence elements through which transcription factors and other genomic regulators act [15]. The formation of active *cis*-regulatory complexes involves the dynamic interplay between sequence-specific DNA binding proteins and nucleosomes and chromatin organizing proteins [16-20]. Binding of multiple sequence-specific regulators within *cis*-regulatory regions results in markedly increased local chromatin accessibility to nucleases, both with respect to flanking genomic regions and to inactive genomic regions generally. For this reason, delineation of DNaseI hypersensitive sites (DHSs) has proven to be a particularly powerful strategy for mapping regulatory DNA in eukaryotic cells [21-24], and recent advances in sequencing technology have enabled DHS mapping at genome scale [25-29]. A salient advantage of this approach is that it permits precise delineation of potential regulatory DNA regions independent of *a priori *knowledge of the particular regulatory factor(s) that may be bound at any given region.

To map the occupancy patterns of specific regulators, chromatin immunoprecipitation (ChIP) has been applied to over 20 developmental transcription factors and RNA polymerase in the blastoderm embryo and, for several factors, at later stages of embryogenesis [30-36]. These studies collectively identify over 20,000 genomic regions occupied to varying degrees by at least one factor, with significant enrichment of known *cis*-regulatory modules (CRMs) among the most highly bound regions [30,31,33]. Recent studies have also mapped binding sites for CTCF and other insulator proteins in *D. melanogaster *embryos [37], as well as origin recognition complex (ORC) proteins in *Drosophila *Kc cells [38]. Both of these features are associated with regions of active, accessible chromatin and nucleosome turnover. Analysis of 53 chromatin-associated proteins localized across the genome in Kc167 cells using DamID has distinguished five major chromatin states, including two active and three repressive states. Active states were enriched in actively transcribed genes, while one repressed state was particularly enriched in genes important for embryonic development.

Here we apply genome-scale, high-resolution mapping of *in vivo *DNaseI sensitivity to define the chromatin accessibility and regulatory DNA landscape of *Drosophila *early embryo development. We mapped DHSs across the *D. melanogaster *genome at five developmental stages (stages 5, 9, 10, 11 and 14) encompassing the transition from a pre-gastrulation (stage 5 blastoderm) to the largely differentiated tissues at stage 14, and in the widely used Kc167 cell line. Our results show that the chromatin landscapes of undifferentiated and more differentiated embryos are similar in terms of the number and distribution of chromatin accessibility and DHSs, with a largely balanced developmental acquisition and loss of DHSs and associated *cis*-regulatory potential. The dynamic chromatin landscape of development is characterized by focused temporally programmed changes occurring at the level of individual DHSs. This contrasts sharply with the wholesale changes in chromatin organization observed between embryos and a static cell line. We were able to associate thousands of developmentally patterned distal DHSs with distinct spatial and temporal expression patterns of linked genes as well as larger regions of chromatin plasticity. Analysis of chromatin remodeling at promoter regions revealed a novel role for classical core promoter sequence elements in directing temporally regulated chromatin architectures. An unexpected feature of the chromatin landscape was the presence of developmentally regulated, localized accessibility and weak DHSs over many protein-coding regions. Subsets of these regions are associated with blastoderm-stage transcription of genes that receive prominent maternal RNA contributions. The results collectively provide a global view of chromatin landscape dynamics during early animal development.

## Results

### Developmental profiling of chromatin accessibility and DHSs

**Figure 1:**
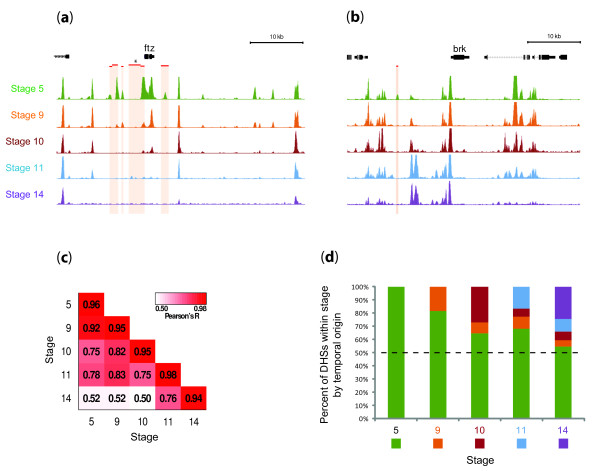
**DHSs exhibit programmed developmental changes (a,b)**. Developmental profiling at *ftz *and *brk *loci. The density of mapped DNaseI cleavages (150-bp sliding window, step 20 bp) is shown for stages 5 (green), 9 (orange), 10 (red), 11 (blue) and 14 (purple) across a 50-kb region of the *D. melanogaster *genome that includes the (a) *ftz *and (b) *brk *genes. Locations of known *cis*-regulatory modules (CRMs) are indicated with red bars and underlying shaded regions. CRMs shown are all known to be active at stage 5 and inactive at later stages except the one indicated with an asterisk, which is a neuronal CRM active after stage 5. Temporally dynamic patterning of chromatin accessibility at DHSs is evident in up- and down-regulation of accessibility during embryo development. **(c) **High reproducibility of DNaseI sensitivity profiles. The pairwise Pearson correlations between DNase I cleavage density datasets from different stages (or between replicates of the same stage, along the diagonal) are indicated in a spectrum from red (extremely high correlation) to white (moderate correlation). The largest differences are observed between stage 14 and earlier stages. **(d) **Developmental propogation of DHSs. Stage 9 DHSs were divided into two groups, those observed at stage 5 and those that arise during the transition from stages 5 to 9. Likewise for stages 10, 11, and 14 the percentages of DHSs are depicted according to stage of temporal origin: stage 5 (green), 9 (orange), 10 (red), 11 (blue) and 14 (purple). The majority of sites (approximately 55%) observed at stage 5 are carried forward through stage 14.

To map DHSs during *Drosophila *embryogenesis and to profile their accessibility within chromatin as a function of time, we collected and pooled cages of *D. melanogaster *embryos at 3, 4, 5, 6, and 11 hours, corresponding to the transition from the cellular blastoderm (stage 5) through the formation of organ primordia (stages 9, 10, and 11) and the beginning of head involution (stage 14). We harvested embryonic nuclei, treated with DNaseI, and isolated small DNA fragments liberated by closely spaced DNaseI cleavages on the same linear chromatin template [39]. To map individual DNaseI cleavages to the genome, DNaseI-released fragments were assembled into sequencing libraries and end-sequenced on an Illumina GA2 instrument [27], yielding an average of approximately 14 million genomic reads per sample that mapped to a unique position within the approximately 118-Mb euchromatic genome [40], resulting in a dense profile of DNaseI cleavage across the genome (Figure 1a,b). These profiles revealed a highly developmentally dynamic chromatin landscape, suggesting tight, programmed regulation of chromatin accessibility during embryo development. Data from each stage were analyzed using a scan-statistic algorithm [28] to delineate accessible chromatin regions defined by significantly increased DNaseI cleavage density (see Materials and methods) within which we identified 45,825 highly significant (false discovery rate (FDR) 1%) and reproducible peaks in DNaseI cleavage density characteristic of DHSs (Table 1; Table S1 and Figure S1 in Additional file 1; Additional file 2). These DHSs collectively (across all stages) cover 6.4% of the euchromatic genome (7.6 Mb), of which an average of 3.5% (4.1 Mb) is DNaseI hypersensitive within any given stage. An additional 13.1% of the genome displays significantly increased DNaseI sensitivity, which is generally found in the regions immediately flanking DHSs (Figure 1; Table S1 in Additional file 1; Additional file 3). The number of DHSs defined at each stage varied approximately 1.3-fold, with the highest numbers observed in stage 5 (Table 1).

**Table 1:** Landscape of *Drosophila *embryo DNase I hypersensitive sites

Stage	Consensus DHSs (FDR 1%)	Percentage of genome
5	30,509	3.8%
9	28,546	3.5%
10	28,318	3.6%
11	28,054	3.5%
14	23,653	3.0%
**All**	**45,825**	**6.4%**

### Balanced developmental restriction and expansion of accessible chromatin

Replicate DNaseI sensitivity measurements from pooled nuclei from each stage were highly reproducible (mean genome-wide correlation for raw tag density R = 0.96; Figure 1c). DNaseI cleavage densities from immediately adjacent stages were also highly concordant, with monotonic decay of correlation between progressively more distant stages (Figure 1c). At the level of DHSs, we observed both strong persistence of DHSs between successive stages, and the appearance of new DHSs (Figure 1d). Of the detected DHSs within stage 14 chromatin, 54.7% were carried forward from stage 5, with the remainder (45%) having originated in stages 9 (4.5%), 10 (6.4%), 11 (9.6%), and 14 (24.5%) (Figure 1d). As such, the developmental restriction of *cis*-regulatory regions marked by DHSs appears to be largely balanced by the synchronous appearance of new elements.

### Genomic distribution and relationship with genic and functional genomic annotations

To assess how comprehensively the chromatin accessibility data illuminated well-documented embryonic regulatory DNA regions, we analyzed 60 previously described experimentally validated CRMs active within blastoderm embryos and known to be bound by multiple transcription factors [33,35,41,42]; 100% of these elements displayed significantly increased chromatin accessibility in stage 5 embryos. We obtained analogous results in a distinct set of CRMs identified initially using ChIP-chip data and tested in transgenic embryos (W. Fisher, A. Hammonds, X.-Y. Li, M.B. Eisen, M.D. Biggin and S.E. Celniker, in preparation). Of the 42 elements active in *in vivo *transgenic promoter experiments at stage 5, 100% exhibited high accessibility in stage 5 chromatin. Additionally, of the 45 CRMs active *in vivo *at stage 14, 70% showed significantly increased accessibility in stage 14 chromatin - a surprisingly high percentage in view of the fact that many later elements are active in only a small fraction of the cells of the embryo (W. Fisher, A. Hammonds, X.-Y. Li, M.B. Eisen, M.D. Biggin and S.E. Celniker, in preparation). The *P*-values for each of these associations are very low (*P *< 1e-16) using either the genome structure correction method or a binomial model.

**Figure 2:**
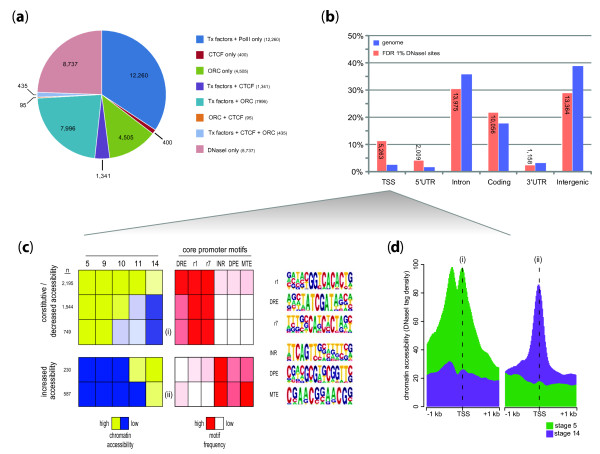
**DHSs overlap orthogonally-measured functional regulatory elements**. (a) DHS locations correlate with functional regulatory sites from orthogonal datasets. Pie chart depicting the percentage of all DHSs identified across all stages in non-coding sequence (*n *= 35,769 at FDR 1%) that overlap the binding locations of other factors: CTCF, ORC, and/or any 1 of 21 developmental transcription factors. **(b) **DHSs are enriched at transcription start sites (TSSs) relative to genomic feature percentage. The bar graph depicts the percentage of all 1% FDR DHSs identified across all stages whose central nucleotides are located within 100 bp of a TSS, or in 5' UTRs, coding sequences, introns, 3' UTRs or between genes (intergenic). **(c) **Core promoter composition directs temporal changes in accessibility of TSSs. The peak in DNase I cleavage density was determined for each stage at the -60 to +40 regions of each promoter, and was clustered using kmeans. The average peak density at each stage and for each cluster is shown at left in a spectrum from yellow (high) to blue (low), forming two metaclusters: one that is constitutively high or exhibits a decrease in accessibility during development (top panels), and another set of promoters that exhibit increasing accessibility during development (bottom panels). For each cluster, the relative enrichments of each of six previously identified core promoter motifs found in each cluster are shown in a spectrum from red (high) to white (low), with the sequence logos for each motif presented on the right. Three motifs, the DNA replication-related element (DREF), r1 and r7, were greatly enriched within constitutive/down-regulated promoters, while the downstream promoter element (DPE), the initiator (INR), and MTE (motif ten element) were enriched in the upregulated promoters. **(d) **Different promoter classes exhibit distinct structural morphologies. Chromatin accessibility in terms of mean DNaseI tag density was plotted within a 1-kb window of the TSS for clusters indicated as (i) and (ii) in panel (c). Chromatin accessibility for stage 5 is shown in green and that for stage 14 in purple. In addition to the developmental profiling of these promoters, (i) shows a distinct double-peaked pattern that is different from the patterns of DNaseI cleavage around other promoter types.

We next assessed the overlap of DHSs (considering all stages collectively) within non-coding regions with occupancy patterns of three classes of genomic regulators (and combinations thereof) defined by ChIP-chip studies: (i) 21 sequence-specific developmental transcription factors plus RNA polymerase II delineated in blastoderm embryos [31,33]; (ii) the insulator protein CTCF [37] profiled in pooled 0- to 12-hour embryos; and (iii) the ORC factor defining origins of DNA replication as profiled in S2 cells [38]. We had shown previously that a majority of the genomic regions accessible at stage 5 overlap regions bound by at least one of the 21 developmental factors or RNA polymerase [32]. Of 35,769 non-coding DHSs from all stages, 27,032 overlapped regions occupied by these factors or by CTCF or ORC (*P *< 10^-16^; Figure 2a). It is notable, however, that the number of DHSs not associated with these occupied regions (*n *= 8,737) is likely considerably underestimated due to the relatively low resolutions of the ChIP-chip factor occupancy assays (approximately a 1.2-kb average for transcription factors, RNA polymerase II, and CTCF, and an average of 3.5 kb for ORC) versus the precision with which DHSs were mapped (average 150 bp), leading frequently to apparent overlap of multiple DHSs within a single factor or ORC occupancy-defined region.

To determine the genomic distribution of DHSs relative to genic annotations, we computed the proportions of DHSs around annotated transcription start sites (TSSs; from -60 to +40), and within 5' and 3' UTRs, protein coding exons, introns, and intergenic regions (Figure 2b). Overall, approximately 12% of DHSs were localized around TSSs, while 31% were found in introns, and 29% in more distal intergenic regions (Figure 2b). DHSs exhibited strong enrichment relative to random expectation around TSSs and 5' UTRs, moderate enrichment over protein coding exons, and relative depletion in intronic and intergenic regions (Figure 2b).

### Distinct combinations of motifs predict early and late promoter accessibility patterns

Evidence has recently emerged that suggests a more complicated and active role for core promoter elements in regulated gene expression [43]. We therefore examined the relationship between core promoter structure (as reflected in the pattern of core promoter motifs) and developmental alterations in core promoter remodeling/accessibility, which is a prerequisite for (though does not necessitate) transcriptional activity. Prior functional studies have extensively characterized several critical core promoter elements, including TATA, the initiator (INR), the downstream promoter element (DPE), and the DNA replication-related element (DRE or DREF) [43]. In addition, six novel core promoter motifs have been defined on the basis of intra-genomic TSS comparisons and evolutionary conservation [44], of which one, MTE (motif ten element), was subsequently shown to facilitate INR-mediated transcription [45].

We therefore first determined the presence (or absence) of the aforementioned ten motifs within the core promoter regions (-60 to +40) defined relative to the annotated TSSs of all *Drosophila *genes. We then related the patterns of core promoter motif occurrence with developmental patterning of chromatin accessibility. This revealed a striking and nearly mutually exclusive relationship between specific sets of core promoter motifs and genes that exhibit constitutive or early promoter chromatin accessibility versus those with late-peaking accessibility (Figure 2c). Genes with either constitutive or early peaking accessibility are significantly enriched for DRE and motifs 1 and 7 (from Ohler *et al*. [43]), whereas genes with late-peaking accessibility are highly enriched for INR, DPE, and MTE motifs.

Changes in promoter motif composition are also accompanied by clear alterations in chromatin structure. While most promoters show a single accessibility peak centered just upstream of the TSS, others with specific motif combinations displayed more complex structures. For example, a subset of early accessible promoters with DRE and Ohler motifs 1 and 7 exhibit a prominent 'camelback' morphology, with a trough located approximately 150 bp upstream of the TSS (Figure 2d). By contrast, promoters with late-onset accessibility and enriched in INR, MTE, and, to a lesser extent, DPE, show single accessibility peaks more closely apposed to the TSS. Taken together, these findings suggest a prominent and previously unappreciated role for the core promoter in developmental patterning of promoter chromatin remodeling.

### Developmentally regulated accessibility at protein-coding exons

We noted that a subset of DHSs overlapped coding exons, prompting us to explore this relationship more fully. In total, we identified 10,056 DHSs that overlapped the protein coding portions of exons in one or more developmental stages (Figure 2b). These elements were predominantly only weakly accessible, with mean DNaseI cleavage density approximately 2.5-fold lower than the mean for all other DHSs, and four-fold lower than average DHSs upstream of TSSs. This finding parallels prior observations of low-level regulatory factor occupancy over protein coding exons [32]. Chromatin accessibility over coding exons displayed prominent developmental regulation, of similar magnitude to DHSs in other genomic regions (Figure S2 in Additional file 1).

We also observed systematic skewing of chromatin accessibility toward the 5' ends of exons and over immediately adjacent 5'-upstream intronic regions. The degree of 5' skewing was strongly correlated with RNA polymerase II occupancy over the exon as measured by ChIP-chip (Figure S3 in Additional file 1). The occurrence of peak DNaseI sensitivity immediately upstream of exons suggests that peri-exonic accessibility patterns may, in fact, reflect the actuation of nearby upstream intronic *cis*-regulatory elements.

### Maternally loaded exons and blastoderm chromatin accessibility patterns

To visualize peri-exonic chromatin accessibility more clearly, we identified all exons of at least 320 bp in length with at least 300 bp of uninterrupted intronic sequence both 5' and 3' to the up- and down-stream intron-exon boundaries (*n *= 4,575 exons). We then computed chromatin accessibility over each peri-exonic region, and clustered these values into four groups reflecting increasing intensity and extent of exonic and exon-proximal accessibility (Figure S4 in Additional file 1). Surprisingly, these accessibility patterns were strongly correlated with the number of exons in each cluster that exhibited elevated RNA abundance (signal >50) between 0 and 2 hours of embryonic development [12]. At this early time point prior to the onset of zygotic transcription, most RNA signal is expected to derive from maternally contributed transcripts [46]. Increased blastoderm chromatin accessibility around maternally loaded exons suggests that these regions may be programmed for rapid early activation following the dissipation of maternal transcripts.

### Extensive plasticity of chromatin domains between embryos and static cell lines

**Figure 3:**
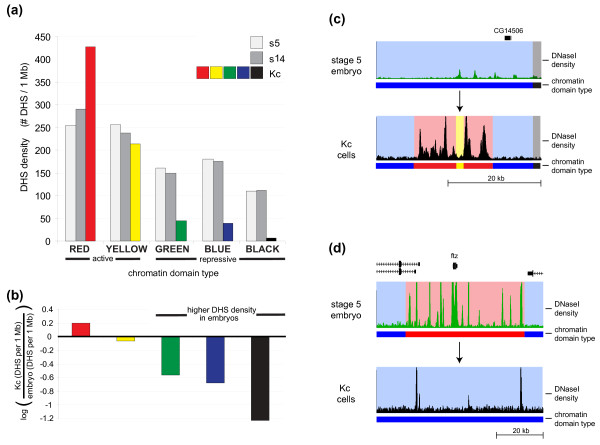
**Chromatin domains of embryonic cells *in vivo *show extensive differences from those in cell cultures**. **(a) **The number of DHSs per megabase is plotted for DHSs from stage 5 embryos (white), stage 14 embryos (gray) and Kc cells for DHSs mapping to each of the five chromatin states annotated in Kc cells (red, yellow, green, blue, and black). A much larger proportion of DHSs in Kc cells map to active chromatin than to repressive chromatin, while DHSs from stage 5 and 14 are divided among the Kc domains. **(b) **The log ratio of embryo to Kc DHSs/Mb shown in panel (a), showing the enrichment of embryonic DHSs at regions that represent repressed chromatin in Kc cells. These enrichments suggest extensive plasticity between the two *Drosophila *systems. **(c) **An example of chromatin that is active in Kc cells but not in embryos. Plotted for stage 5 embryos and Kc cells is the DNaseI density and colored chromatin state (red and yellow = active, blue and black = repressive). **(d) **An example of chromatin that is active in embryos but not in Kc cells.

The extent to which chromatin domains are plastic between embryonic stages, let alone between different *D. melanogaster *cell systems, is unknown. To place the developmental dynamics observed between embryonic stages into context, we examined the complement of 19,378 FDR 1% DHSs observed in Kc167 cells [47]. We then compared the distribution of Kc167 DHSs with the five chromatin states defined by Filion *et al*. [48]. Kc167 DHSs were heavily skewed towards regions of active chromatin delineated by occupancy patterns of diverse chromatin proteins (Figure 3a). By contrast, DHSs from stage 5 and 14 embryos were significantly more enriched within repressive Kc167 chromatin domains, and depleted from one subclass ('Red') of active Kc167 chromatin (Figure 3a,b). These results suggest extensive differences in the chromatin compartments between developing embryos and temporally static cell lines. Indeed, we identified numerous DHS-dense regions in Kc167 cells that corresponded to active Kc chromatin, yet were inaccessible in embryos (Figure 3c). Conversely, we found numerous DHS-dense domains in embryos that mapped within repressed chromatin in Kc cells (Figure 3d). Kc167 DHSs falling into repressive chromatin domains were markedly enriched for suppressor of hairy wing (Su(hw)) motifs (MEME *P *< 1e-64, TOMTOM *P *< 1e-7).

### Stereotyped temporal patterns of chromatin accessibility at regulatory DNA

Many DHSs are characterized by significant stage-to-stage variability in DNaseI sensitivity, and show graded, monotonic increases or decreases in accessibility along a temporal axis (Figure 1; Figure S1 in Additional file 1). For example, the blastoderm-specific CRM marked by a DHS downstream of *ftz *[49] is accessible at stage 5 but not stages 9, 10, 11 and 14 (Figure 1). Also, neuronal enhancer active in late embryogenesis [50] first becomes accessible at stage 11 (Figure 1).

**Figure 4:**
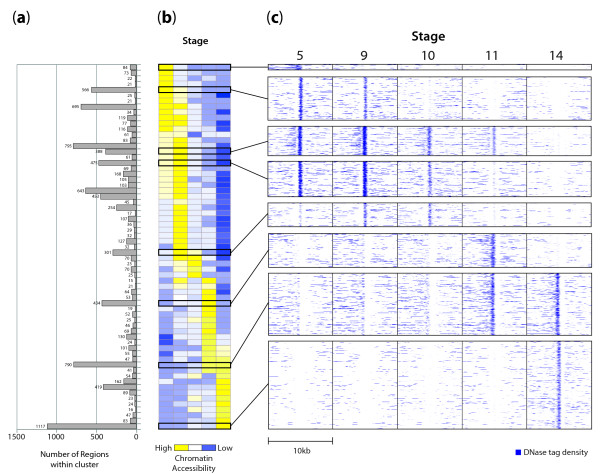
**Chromatin accessibility patterns at developmentally dynamic elements**. Developmentally dynamic elements (DDEs; see text for definition) were clustered according to quantitative accessibility patterns, and ordered according to the time of peak accessibility. **(a) **Number of DDEs in each cluster. **(b) **Average accessibility at each stage for all regions within the cluster. Each row in panels (a) and (b) represents a distinct cluster (*n *= 65). **(c) **Selected clusters from (b), which are expanded to the resolution of individual elements, wherein each pixel row depicts DNaseI sensitivity (raw tag density, highest in yellow) in a 10-kb window around each DDE in the cluster.

To delineate systematically such developmentally dynamic elements (DDEs) showing either stage-specific or temporally graded alterations in chromatin accessibility, we developed a robust quantitative method for identifying regions showing similar temporal chromatin accessibility patterns (Materials and methods; S. Thomas, S. Neph, A. Reynolds, J.A. Stamatoyannopoulos, in preparation). We identified 11,014 DDEs collectively covering approximately 1.5% of the euchromatic genome, to which we applied an unsupervised clustering approach [51], yielding 65 clusters each comprising elements with nearly identical temporal accessibility profiles (Figure 4). The majority of DDEs could be partitioned into two major groups - those showing peak accessibility in early development (*n *= 4,166) versus those with peaking accessibility in stages 11 to 14 (*n *= 4,431) (Table 2). A separate group comprised elements accessible only at a single stage (*n *= 1,940), with stage 14-specific elements accounting for the significant majority (75%, 1,446 out of 1,940). A small proportion of DDEs (*n *= 283) displayed undulating accessibility patterns, such as diminished or enhanced accessibility during the middle stages. Overall, the largest fraction of the 65 temporal patterns we defined encompassed the transition from stage 11 to 14, likely reflecting the extensive differentiation of cell types that occurs between these stages [2].

**Table 2:** Number of developmentally dynamic elements belonging to different temporal pattern classes

Temporal pattern class	Number of DDEs	**Percentage of total**^**a**^
Stage 5 specific	193	1.75%
Stage 9 specific	50	0.45%
Stage 10 specific	21	0.19%
Stage 11 specific	423	3.84%
Stage 14 specific	1446	13.13%
Early	4431	40.23%
Late	4166	37.82%
Mixed	283	2.57%

^a^The percentage is out of the total of 11,014 DDEs identified.

### Developmentally dynamic elements are conserved and cluster into dynamic domains

**Figure 5:**
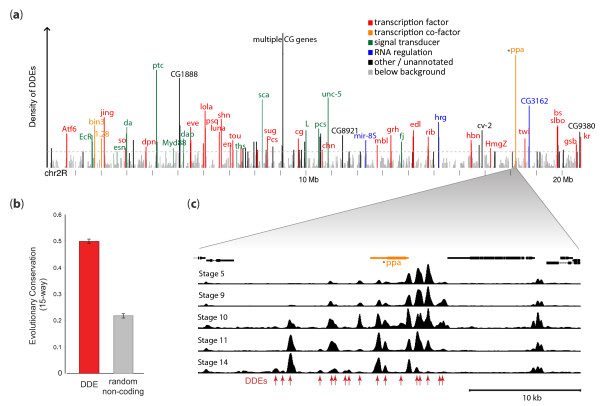
**Developmentally dynamic domains are enriched in regulatory genes**. **(a) **Density of DDEs plotted across chromosome 2R. Peaks in DDE density above a statistical background (dotted grey line) were labeled by associated gene name and colored by gene ontology (GO) category. **(b) **The median phastcons conservation score (and 95% confidence intervals) for all DDEs that map to non-coding locations is shown alongside the median and intervals calculated for randomly chosen non-coding sites within the genome. **(c) **DNaseI tag density across an approximately 30-kb region of chromosome 2R around ppa, indicated with an asterisk in (a,c), illustrating an exemplary developmentally dynamic domain composed of clustered DDEs (red arrows).

We next examined the distribution of DDEs along the genome. Plotting the density of DDEs as a function of genomic position revealed a strikingly inhomogeneous distribution, with frequent dense clustering of DDEs (Figure 5). We also found that DDEs active predominantly in either earlier or later developmental stages were highly clustered with similarly patterned elements, over a median range of 39 kb (Figure S5 in Additional file 1). DDEs are generally strongly evolutionarily conserved, indicating their functional importance (Figure 5b).

By clustering DDEs along the genome, we delineated 890 developmentally dynamic domains (DDDs) comprising significant clusters of DDEs with shared temporal profiles (Additional file 4). These domains ranged in size from 10 kb to 70 kb (mean 27 kb), and collectively encompassed 11.6% of the euchromatic genome (including DDEs as well as the intervening inaccessible regions). It is notable that some DDDs contain not only a cluster of DDEs with similar temporal profiles, but may also encompass interspersed constitutive elements that do not show temporal bias, or, more rarely, isolated elements that may show a temporal bias differing from the domain as a whole.

### Developmentally dynamic domains mark developmental regulatory genes

We next examined how DDDs were distributed with respect to genes, and specifically if there were particular classes of genes that were enriched within DDDs generally (that is, irrespective of the particular temporal profile of the DDE). We observed a striking relationship between domains with high DDE density and genes encoding transcription factors, transcriptional co-factors, signal transducers, or other regulatory genes (Figure 5a). We also observed a specific concentration of developmental regulators (versus generic transcriptional regulators) within such DDDs. For example, the 200 domains with the highest density of DDEs contain, among other regulators, 28 transcription factors, of which 24 are well-studied developmental regulators. We also observed a quantitative relationship between DDE density and transcription factors, with lower DDE density associated with a lower proportion of transcription factors among the overlapped genes (Figure S6 in Additional file 1).

These results indicate that DDEs are enriched in CRMs important for controlling the regulators important for development, and suggest that the DDEs within high-density domains may encode CRMs controlling many developmental regulators. This indication is further supported by the observation that 85% of a set of 53 spatially patterned CRMs active at diverse points across embryogenesis (including many late elements; W. Fisher, A. Hammonds, X.-Y. Li, M.B. Eisen, M.D. Biggin and S.E. Celniker, unpublished data) coincide with a DDE, in spite of the fact that DDEs cover only 1.5% of the genome. Surprisingly, the few remaining regions with high DDE density that were not associated with transcriptional regulators were mainly associated instead with genes of unknown function, including many regions among the top 10% in DDE density. This suggests that these genes may, in fact, encode as-yet-uncharacterized developmental regulators.

### Spatio-temporal gene expression patterns parallel developmentally dynamic chromatin

**Figure 6:**
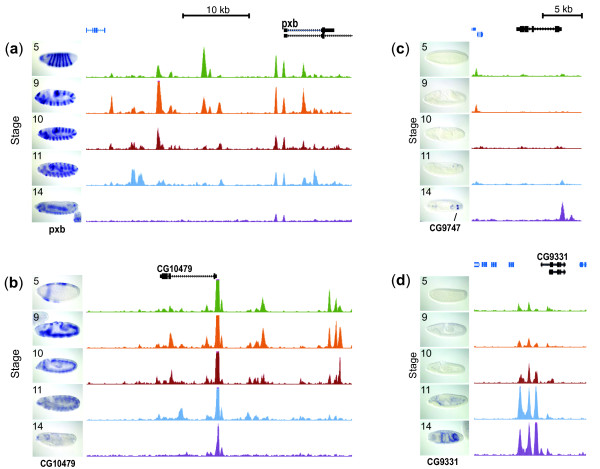
**DNaseI patterns correlate with *in situ *spatio-temporal expression patterns and demonstrate the high sensitivity of the assay**. For each panel, DNAse I tag densities for four genes at stages 5, 9, 10, 11 and 14 are shown in green, orange, red, blue, and purple, respectively. On the left of the accessibility plots for each stage are images from the BDGP *in situ *mRNA expression database of that gene during the relevant stage. **(a,b) **Decreases in chromatin accessibility near the *pxb *(a) and *CG10479 *(b) genes were associated with concomitant changes in spatio-temporal expression of the genes *in vivo*. **(c,d) **Increases in chromatin accessibility through development at the *CG9747 *(c) and *CG9331 *(d) genes were associated with increases in expression of the gene *in vivo*. Even though a relatively low percentage of cells are expressing the *CG9747 *gene at the latest stage in (c), an associated change in chromatin accessibility is still reflected in the chromatin accessibility profile, demonstrating the sensitivity of the DNaseI assay.

We next determined how the temporal accessibility profiles of DDEs were related to the spatial and temporal expression patterns of nearby genes. For each DDE we retrieved expression pattern annotations that were associated with the gene whose TSS was nearest the DDE. The expression pattern information was derived from a large scale effort by the Berkeley *Drosophila *Genome Project (BDGP) that uses *in situ *mRNA localization followed by manual annotation of the tissues and stages that approximately 6,000 genes are expressed in [14]. Within each of the 64 temporal clusters of DDE accessibility, the probability of enrichment of each annotated spatio-temporal expression term was calculated (Figure S7 in Additional file 1). We observed a clear relationship between chromatin accessibility changes and mRNA expression pattern. For example, the clusters of DDEs whose chromatin accessibility is greatest in the pregastrula (stage 5) embryo tend to be associated with nearby genes that are expressed in patterns at this stage. Conversely, the DDE clusters with highest accessibility at stage 14 tend to be nearest genes expressed at this stage. Comparison of the accessibility profiles and the mRNA expression patterns of four individual genes confirms this trend (Figure 6). Interestingly, even clusters with relatively similar temporal profiles (for example, the left-most ten columns of Figure S7 in Additional file 1) show marked differences in the specific subsets of embryo cells in which their associated genes are expressed, suggesting that the DDE clusters represent sets of regulatory elements that share some communality in their control.

Not all of the approximately 300 BDGP gene expression annotation terms are significantly enriched in the accessibility clusters. Largely late tissue-specific expression terms are missed. This is not unexpected, however, as in stage 14 embryos there are many more tissues/annotation terms and these typically each represent a smaller percentage of the embryo. Thus, these terms are less likely to be captured as statistically significant in our analysis. We suggest our DDE clusters could represent a broad temporal mode of control, one that is in addition to the fine-grained tissue patterning captured in the BDGP's annotations. In which case, in the late embryo in particular, each DDE cluster could include genes that are each expressed in different tissues, but which share a common temporal control mechanism.

## Discussion

A longstanding question surrounding animal development is whether the transition from an undifferentiated pregastrula to a late embryo entails the sequential restriction or an expansion of the *cis*-regulatory landscape. We have mapped millions of individual *in vivo *DNaseI cleavages to produce the first genome-wide maps of the *Drosophila *chromatin accessibility landscape during development from an undifferentiated blastoderm to a highly differentiated late embryo. DHSs are the *sine qua non *of active *cis*-regulatory elements, and the fact that 100% of well-defined blastoderm and 75% of later stage *cis-*regulatory modules coincide with DNaseI-accessible elements suggests that a reasonably comprehensive mapping of accessible regulatory DNA regions active during the surveyed stages has been obtained. This is further supported by the extensive overlap of DHSs with mapped occupancy sites for blastoderm transcriptional regulators [32], the insulator factor CTCF, and DNA replication origins marked by the ORC complex.

The very high proportion of Kc167 DHSs localized within active chromatin domains defined by occupancy patterns of dozens of chromatin proteins [48] contrasts sharply with the >50% of embryonic DHSs that map within genomic domains designated as repressive in Kc cells. The presence of wholesale differences in chromatin compartmentalization between embryos and static cell lines highlights the dynamism of the chromatin landscape across developmental or differentiation gradients.

During early development, chromatin dynamics are exemplified by the widespread developmental patterning of DHSs, which appears to be largely balanced between the extinction of DHSs formed in earlier stages, and the timed appearance of new sites during the progress of development (Figure 1b). DDEs are: (i) evolutionarily conserved; (ii) clustered along the genome; (iii) particularly enriched around genes encoding transcriptional regulators; and (iv) associated with specific spatiotemporal expression programs. In the case of promoters, we identified specific sequence features associated with temporal down- versus up-regulation of chromatin accessibility. Unexpectedly, localized and developmentally regulated chromatin accessibility was also found over protein-coding sequences (albeit weakly), where it closely paralleled both RNA polymerase II occupancy as well as RNA abundance measured prior to the onset of zygotic transcription (0 to 2 hours). The observed blastoderm chromatin patterns may therefore reflect 'programming' of genes or protein-exons for rapid transcription activation coinciding with the dissipation of maternal RNA contributions.

### Developmentally dynamic elements and domains

Developmental patterning of DHSs is highly stereotyped, with large cohorts of genomically dispersed sites displaying almost identical patterns of quantitative change during development. These cohorts are frequently associated with genes that fall into specific combinations of spatial and temporal expression classes. This suggests that the collective action of diverse developmental regulators results in limited complexity at the level of chromatin accessibility, which likely results from shared aspects of regulation among similarly behaved DHSs.

It has long been known that small groups of neighboring genes, such as the Bithorax and Antennapedia complexes [4,52], exhibit temporally correlated expression. More recent work has shown that clustering of genes with related gene expression patterns is common and is also associated with clustered binding of chromatin organizing proteins, patterns of histone modification and the tendency of regions to be physically close to one another in the nucleus [53-56]. The clustering of DDEs showing similar temporal patterns into 10- to 70-kb domains thus likely reflects the coordinate regulation of individual genes by groups of different CRMs with similar temporal activity profiles.

A notable feature of the data is the decline in the total number of 1% FDR DHSs detected during embryogenesis (from approximately 30,000 in stage 5 to approximately 20,000 in stage 14; Table 1), which is paralleled by a substantial increase in the appearance of stage-specific elements. Indeed, stage 14 elements account for 75% of stage-specific DDEs. The appearance of novel elements at stage 14 is consistent with the emergence of specialized cell populations and the dramatic increase in spatially and temporally patterned gene expression at this stage versus blastoderm [10,11,13,14]. Because our DNaseI experiments measured average accessibility for the whole embryo, it is likely that we have failed to detect some elements that are accessible in only a very small percentage of the cells. However, it seems unlikely that this technical failure is the sole explanation. Instead, other biological explanations are suggested by observing the fate of chromatin accessibility at stage 5 DHSs as development progresses. A large fraction of stage 5 DHSs exhibits gradually fading accessibility, as illustrated in Figures 1, 4c, and 5c, rather than suffering a rapid decline. One interpretation of this pattern is that the regulatory factors required for the maintenance of accessibility are diminishing in abundance. However, the number of sites affected is quite large, and is balanced by a large number of unaffected sites that would have been expected to be affected if general transcriptional factors were involved. Another explanation is that diminishing accessibility of stage 5 DHSs is a consequence of sequential cellular restriction of elements that may be accessible in early stages, but only destined for functional activity at later stages. Indeed, such pre-potentiation of chromatin accessibility at *cis*-regulatory DNA prior to the actual function of an element in control of transcription has long been described [16-20], suggesting that this is at least part of the reason why there are similar numbers of accessible regions in early and late embryos despite the fact that the total fraction of active CRMs is much higher later.

### Developmentally patterned accessibility over promoter regions

Our results suggest that localized developmental patterning of chromatin accessibility at promoter elements is related, at least in part, to the structure of the core promoter. The core promoter is a universal mediator of transcription initiation by RNA polymerase II in eukaryotic cells, and has traditionally been regarded as a downstream target of cell- or condition-specific regulatory signals rather than as an intrinsic determinant of such regulation [57]. However, limited evidence from select genes is emerging that suggests a more complicated and active role for core promoter elements in regulated gene expression [43]. Prior functional studies have extensively characterized several critical core promoter elements including TATA, INR, DPE, and DRE/DREF [43]. In addition, six novel core promoter motifs have been defined on the basis of intra-genomic TSS comparisons and evolutionary conservation [44], of which one (MTE) was subsequently shown to facilitate INR-mediated transcription [45]. We found that distinct complements of these core promoter elements were associated with early (DRE/r1/r7) or late (INR/DPE/MTE) appearance of chromatin remodeling over the promoter region, and with the presence of distinct promoter chromatin accessibility morphologies. These results highlight broader effects of core promoter architectures, exposing a novel connection between core promoter architecture and the regulation of promoter chromatin remodeling.

## Conclusions

The dynamic chromatin accessibility landscape of *Drosophila *early development exposed by our studies should provide a rich resource for future analyses. We have highlighted thousands of novel elements that appear to have the properties of *cis*-regulatory DNA, and which can be further explored both experimentally and computationally. The connection between the developmental timing of promoter chromatin remodeling and core promoter architecture identifies potential roles of previously unassigned motifs, and suggests links between established elements that can be tested experimentally. The correlation between peri-exonic chromatin accessibility patterns and pre-zygotic RNA abundance suggests a novel avenue for exploring the transition from maternal to zygotic transcription. Finally, the dramatic differences in chromatin compartmentalization between early embryos and model cell lines highlights the essential plasticity of the chromatin landscape.

## Materials and methods

### Nuclear isolation and DNaseI digestion

Nuclei from *D. melanogaster *embryos were isolated as described previously [58] and treated with DNAse I as previously described with some modifications. Briefly, the embryos were collected in population cages for 1 hour and allowed to develop to stage 5 (2 hours 10 minutes), 9 (3 hours 20 minutes), 10 (4 hours), 11 (5 hours 40 minutes), or 14 (9 hours 50 minutes) as desired at standard conditions. The embryos were dechorionated and homogenized in 5 ml cold buffer A (15 mM Tris HCl, pH 8.0, 15 mM NaCl, 60 mM KCl, 1 mM EDTA, 0.5 mM EGTA, 0.5 mM spermidine) containing 0.5 mM spermine, 0.5 mM dithiothreitol, and 1 mM phenylmethanesulfonylfluoride, for each gram of embryos by using a motor-driven dounce homogenizer. The homogenate was passed through Miracloth, and further homogenized using a dounce homogenizer with pestle B, for five to six strokes, and then 10% NP-40 was added drop-wise to a final concentration of 0.5% with gentle mixing. The nuclei samples were centrifuged in 1.5 ml aliquots in a micro-centrifuge at 3,000 rpm for 3 minutes at 4°C, and the nuclei pellet was washed with buffer A.

Kc167 cells were cultured in Schneider's medium supplemented with 10% heat-inactivated fetal bovine serum at 25°C in a humidified incubator. To isolate nuclei from Kc167 cells, cells were resuspended in buffer A with 0.025% IGEPAL for 5.5 minutes, the nuclei were pelleted in a micro-centrifuge at 3,000 rpm for 3 minutes at 4°C, and the nuclei pellet was washed with buffer A.

Nuclei from embryos and Kc167 cells were purified and treated with DNaseI as previously described [39] with some modifications. After being resuspended in a small volume of buffer A, the number of nuclei was determined, and 50 × 10^6 ^to 70 × 10^6 ^of the pooled nuclei were used in each DNAse I digestion reaction. The DNAse I digestion was carried out by incubating the nuclei with the indicated amount of DNAse I in 2.5-ml pre-equilibrated digestion buffer (buffer A plus 75 mM NaCl and 6 mM CaCl_2_) for 3 minutes at 37°C, and the reactions were stopped by the addition of 2.5 ml of the stop solution containing 50 mM Tris HCl, pH 8.0, 100 mM NaCl, 0.1% SDS, and 100 mM EDTA. The samples were then treated with Proteinase K, and extracted once with phenol/chloroform. Next, the DNA in the samples was fractionated through a sucrose gradient, and fragments ranging from 100 to 400 bp in size were isolated and an Illumina Genome Analyzer I was used to generate sequence tags for each sample. As described previously [27], the sequencing tags were used to map an average of 13.4 million DNAse I cleavage events per sample to *D. melanogaster *genomic sequence. The pairs of replica samples used to analyze stages 5, 9, 11 and 14 were taken from the same collections of staged embryos (one collection per stage), and the samples were divided in two after nuclei were purified but prior to DNaseI digestion. For stage 10, the replica samples were derived from different embryos collected on different days.

### Delineation of DNAse I accessible regions and DNase hypersensitive sites

To identify regions of enriched accessibility, the number of tags within a 250-bp scanning window was compared to the expected number of tags based on a binomial model of the surrounding 50 kb to determine an enrichment z-score. Accessible regions were defined as collections of adjacent tags with z-scores greater than T where the number of background (random) regions with z ≥ T represent 1% of the number of experimental regions with z ≥T (that is, a 1% FDR control).

DNAse I tag density genome-wide was calculated by dividing the genome into 20-bp bins and adding the number of tags within a 150-bp window around each bin. The density scores were then used to identify peaks in accessibility within accessible regions, with each 150-bp peak being designated a DHS. The peak detection method allowed multiple DHSs per accessible region.

For each stage of embryonic development examined, two replicates were performed and the accessible regions for each replicate were intersected to yield a set of 'replicate-concordant' accessible regions (Tables S1 and S2 in Additional file 1). These represent very conservatively defined sets of accessible regions that were found in both replicates. The DHSs from each replicate were retained if they overlapped a DHS from another replicate by 75 bp or more (Table 1; Additional file 2). The union of non-intersecting DHSs from each stage constitute the final DHS list.

### Conservation of DDEs relative to random genomic locations

Using the 12-way phastcon conservation scores [59] the average conservation score across each DDE (that did not overlap a coding sequence) was calculated, and then the total distribution of scores was determined. An equal number of random non-coding sequence positions were selected with equal sizes to the DDE pool and the average conservations were calculated to build the distribution of conservation at random genomic locations.

### Identification of developmentally dynamic elements

Rank expectation was developed as a general method of identifying statistically significant differences between two matching whole-genome datasets (S. Thomas, S. Neph, A. Reynolds, J.A. Stamatoyannopoulos, manuscript in preparation). To identify all locations in dataset A that show increased signal over dataset B, the 20-bp bin scores in B are first ranked from low to high. Then A is sorted by the order of elements obtained from sorting B to achieve A'. That is to say that if the 675th bin in B has the lowest value in the entire dataset, then the 675th bin of A will be listed the first bin in A'. If A and B represent close replicates, then all of the re-ranked bins in A' will have neighbors with approximately equal scores; however, if there is a large difference at a particular site where the signal at B is low and the signal at A is high, then that location will appear out of place in A'. The probability that the score at a bin is drawn from the same distribution as its neighbors is determined from the Gaussian z-score from a local window of scores around each bin in A', since the median absolute variation (mad) around the median in these local windows can be approximated by a normal distribution. Rank expectation was performed on each replicate and in each polarity of comparison. Because two replicates of each stage were performed, for each location there were four measurements indicating whether or not it showed significant enrichment in a given stage over another stage. A bin was said to show 'consistent enrichment' if three or more of these measurements indicated enrichment after controlling for multiple testing using a Benjamini-Hochberg FDR control [60]. Finally, the pairwise enrichment comparisons were tabulated and bins that displayed specific temporal patterns were identified. Bins that showed stage 5-specific chromatin structure, for example, were easily identified from the data as showing consistent enrichment in stage 5 over all other stages examined. Individual 20-bp bins that exhibited change and were adjacent to neighboring bins with similar patterns were merged together to form larger regions defined as DDEs.

### Identification of developmentally dynamic domains

The number of base pairs covered by all DDEs within 10 kb of each 20-bp genomic bin was calculated. A resampling method was used to assess the statistical significance of peaks in DDE density. For each resample, a binomial model was used to draw a number of DDEs that randomly mapped to a particular 10-kb window of the genome. For each randomly mapped DDE a size was drawn from the density of DDE sizes and the DDE density was calculated for the hypothetical window. Ten million bootstraps were used to estimate the probabilities associated with DDE density scores. Peaks in DDE density with probabilities beyond the significance threshold set by Benjamini-Hochberg FDR control [60] at α = 0.05 were defined as DDDs (Additional file 4). The nearest gene to each domain was identified and any relevant gene ontology categories for those genes were identified.

Likewise, the DDE density was calculated for each TSS in the genome [61]. The genes were ranked by DDE density scores and broken into 200-gene bins. The percentage of genes in each bin that were transcription factors was then calculated.

### Analysis of similarly-patterned DDEs

To address the clustering of similarly patterned DDEs (Figure S5 in Additional file 1), the five-dimensional (5 stage) density data for each DDE was put through a simple dimensionality reduction process that generated a single score that represented whether the chromatin structure was weighted towards openness early in development, late in development or consistently distributed. For each genomic location (i) that showed an enrichment, ρ_i _was calculated as follows:

where d_s,i _is the tag density at stage s and location i, and w_s _is an arbitrary weight assigned to each stage:

Thus, if a particular site shows stage 5-specific chromatin, the resulting score would be a large positive number, and if the site was constitutive, then ρ_i _would approach 0.

To determine the degree to which neighboring DDE tended to have similar patterns of accessibility through development, the ρ_i _from adjacent DDEs were compared using a binomial model. If both DDEs exhibited early or late patterns, that constituted a successful Bernoulli trial. The number of observed successes between adjacent DDEs was compared to the binomial distribution to determine a probability of seeing that number of successes randomly. DDEs were then compared to their neighbor's neighbor (two DDEs away) and then to DDEs separated by three DDEs, and so on, all under the background binomial model assuming equal probability of success or failure.

### Overlap of DNAse accessible regions and DDEs with active CRMs

We analyzed a set of 53 CRMs that were initially identified based on ChIP-chip data and subsequently shown to be active at different stages of embryogenesis (W. Fisher, A. Hammonds, X.-Y. Li, M.B. Eisen, M.D. Biggin and S.E. Celniker, unpublished data). The overlap between these sequences and DHSs and DDEs was determined as the number of CRMs that overlapped an accessible region or a DDE by at least 1 bp, divided by the total number of CRMs active at any analyzed stage. The statistical significance of this association was measured using the Genome Structure Correction tool [25] and by a binomial model in R [62].

### Correlating DDEs with spatio-temporal expression patterns of nearby genes

The 64 clusters of DDEs were mapped to the nearest gene, and the BDGP mRNA *in situ *expression terms for all of the unique genes were identified [14]. For each of the approximately 300 expression terms a hypergeometric model was used to determine the probability of choosing 'b' genes with that pattern given that there are 'B' total genes with that pattern out of 'N' total genes in the genome and that 'n' genes were drawn without replacement.

### Analysis of core promoter elements

The peak DNAse I cleavage density was calculated for the core promoters (-60 to +40) of each gene from the release 4.3 version of the *D. melanogaster *genome obtained from FlyBase [63]. Using the motif scanning tool FIMO from the MEME package [64], the sequences of the -60 to +40 promoter regions were then scanned for the presence (*P *< 0.0005) of one or more of the ten motifs previously found at core promoters [44]. Promoters were then clustered (k-means) into ten groups comprising similar accessibility profiles. These groups formed three meta-clusters: one exhibiting constitutive accessibility (41% of TSSs); a second with down-regulated accessibility (44%); and a third with up-regulated accessibility (15%). For each cluster, and for each of the ten motifs, the percentage of promoters with the given motif was calculated in order to gauge differences in motif enrichment between clusters.

### Analysis of peri-exonic chromatin accessibility patterns

The peak DNAse I cleavage density was calculated for each 20-bp increment within a 300-bp window around each DHS exon obtained from the release 4.3 version of the *D. melanogaster *genome [63]. The density values were aligned by direction of transcription through the exon and were then aligned separately by the 5' exon boundary and the 3' exon boundary. The total list of exons was then filtered to identify the approximately 4,500 exons whose nearest exon was at least 300 bp away from both the 5' and 3' boundary and whose total exon length was greater than 600 bp. To characterize differences in transcription among exons with different DNAse I cleavage patterns, the DNAse I cleavage densities across the 5' exon boundaries were separated into four kmeans-derived clusters. The average expression [12] over each exon between 0 and 2 hours was calculated. Within each cluster, the percentage of exons with elevated expression (signal >25) was calculated.

### Data availability

All sequence data have been deposited in the NCBI Short Read Archive (SRA) under the following accession numbers: [SRA:SRP002474.1, SRA:SRX020691.4, SRA:SRX020692.1, SRA:SRX020693.1, SRA:SRX020694.1, SRA:SRX020695.1, SRA:SRX020696.1, SRA:SRX020697.1, SRA:SRX020698.1, SRA:SRX020699.1, SRA:SRX020700.1, SRA:SRX041410].

## Abbreviations

BDGP: Berkeley *Drosophila *Genome Project; bp: base pair; ChIP: chromatin immunoprecipitation; CRM: *cis*-regulatory module; DDD: developmentally dynamic domain; DDE: developmentally dynamic element; DHS: DNase I hypersensitive site; DPE: downstream promoter element; DRE/DREF: DNA replication-related element; FDR: false discovery rate; INR: initiator; MTE: motif ten; ORC: origin recognition complex; TSS: transcription start site; UTR: untranslated region.

## Competing interests

The authors declare that they have no competing interests.

## Authors' contributions

ST, XL, JAS and MDB conceived and designed the experiments and analyses and wrote the paper. XL, PJS, TC and EG performed the wet laboratory experiments. ST, RS, RT, JAS and MDB analyzed the data. WF, AH and SC characterized CRM activity. All authors read and approved the final manuscript.

## Supplementary Material

Additional file 1 **Supplementary tables and figures**. Click here for file

Additional file 2 **FDR 1% DHSs in euchromatic DNA for stage 5, 9, 10, 11 and 14 embryos (related to Figures **1**and **2**)**. Genome coordinates of the 1% FDR DHSs at stages 5, 9, 10, 11 and 14. Click here for file

Additional file 3 **FDR 1% accessible regions in euchromatic DNA for stage 5, 9, 10, 11 and 14 embryos (related to Figures **1**and **2**)**. Genome coordinates of the intersection of 1% FDR accessible regions at stages 5, 9, 10, 11 and 14. Click here for file

Additional file 4 **Developmentally dynamic domains in euchromatic DNA**. Genome coordinates of DDDs from Figure 4. Click here for file
