# Supplementary material for: Dynamic reprogramming of chromatin accessibility during Drosophila embryo development
Source: Genome Biol. 2011 May 11;12(5):R43. doi: 10.1186/gb-2011-12-5-r43 (PMC3219966; doi:10.1186/gb-2011-12-5-r43)
Supplement: Additional file 1 — Supplementary tables and figures. [file gb-2011-12-5-r43-S1.PDF]

TABLE S1. Landscape and Stage-selectivity of *Drosophila* embryo accessible regions, related to Figures 1 and 2.

Stage	Consensus accessible regions (FDR 1%)	% of Genome
5	17,022	9.6%
9	12,361	7.5%
10	16,502	8.7%
11	14,563	8.6%
14	15,510	7.0%
**All**	**28,238**	**16.6%**

FIGURE S1. **Developmental profiling at two genomic scales . (a)** The density of mapped DNaseI cleavages (150bp sliding window, step 20bp) is shown for stages 5 (green), 9 (orange), 11 (blue) and 14 (purple) across a 50 kb region of the *Drosophila melanogaster* genome that includes the *ftz* gene. Two technical replicates are shown for each stage. Regions of significantly (FDR 1%) increased DNaseI-accessibility and DNaseI hypersensitive sites (DHSs) are shown under DNaseI tag density profiles for each stage. Locations of known *cis*-regulatory modules (CRMs) are indicated with red bars and underlying shaded regions. CRMs shown are all known to be active at stage 5 and inactive at later stages except the one indicated with a *, which is a neuronal CRM active after stage 5. **(b)** Data for a ~400 kb region around the *Fmr1* gene are shown using the same conventions as panel (a). Temporally dynamic patterning of chromatin accessibility at DHSs is evident in up-regulation and down-regulation of accessibility during embryo development.

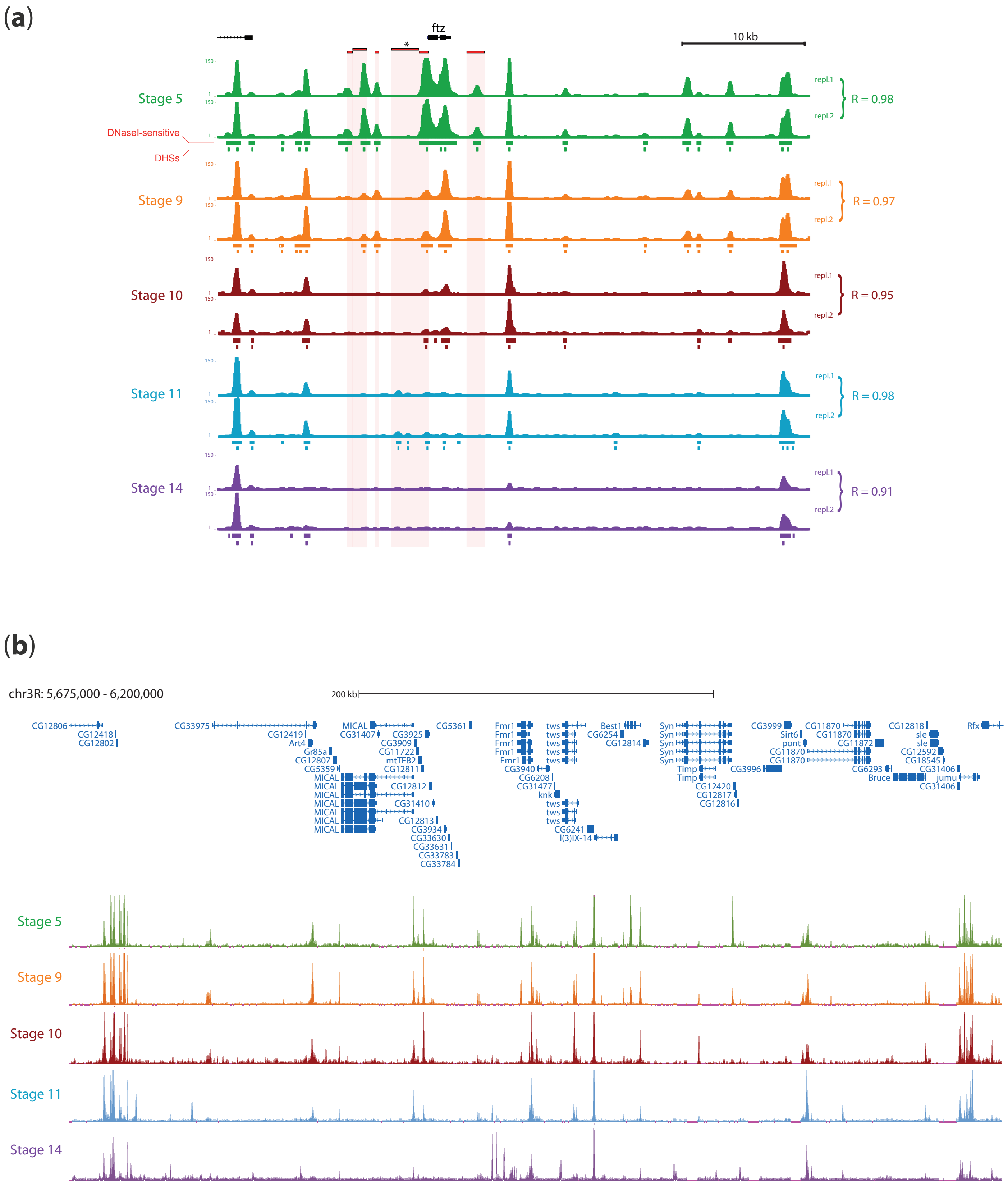
(a)
ftz
*
10 kb
Stage 5
Stage 9
Stage 10
Stage 11
Stage 14
DNaseI-sensitive
DHSs
150
1
repl.1
repl.2
R = 0.98
R = 0.97
R = 0.95
R = 0.98
R = 0.91
(b)
chr3R: 5,675,000 - 6,200,000
200 kb
CG12806
CG12418
CG12802
CG33975
CG12419
Art4
Gr85a
CG12807
CG5359
MICAL
CG31407
CG3925
CG3909
CG11722
mtTFB2
CG12811
CG12812
CG31410
CG12813
CG3934
CG33630
CG33631
CG33783
CG33784
CG5361
Fmr1
CG3940
CG6208
CG31477
knk
tws
CG6241
l(3)IX-14
Best1
CG6254
CG12814
Syn
Timp
CG12420
CG12817
CG12816
CG3999
CG3996
Sirt6
pont
CG11870
CG11872
CG6293
Bruce
CG12818
sle
CG12592
CG18545
CG31406
jumu
Rfx
Stage 5
Stage 9
Stage 10
Stage 11
Stage 14

FIGURE S2. DHS are developmentally regulated. Stage 5 DHS were divided into 6 classes based on the location of the central peak within the different genomic feature types: transcription start site (TSS), 5' untranslated region (UTR), protein coding-sequence (CDS), intron, 3' UTR, and intergenic. The Pearson correlation between peak DNaseI tag densities from the different stages at the stage 5 DHS are shown for each genomic feature. There is a consistent decline in correlation for each feature type from stage 5 through development, indicative of developmental patterning **.**

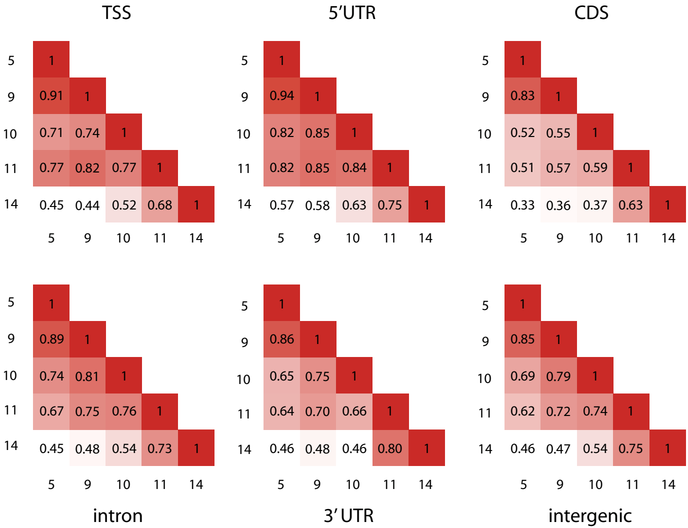
TSS
5'UTR
CDS
5
9
10
11
14
1
0.91 1
0.71 0.74 1
0.77 0.82 0.77 1
0.45 0.44 0.52 0.68 1
1
0.94 1
0.82 0.85 1
0.82 0.85 0.84 1
0.57 0.58 0.63 0.75 1
1
0.83 1
0.52 0.55 1
0.51 0.57 0.59 1
0.33 0.36 0.37 0.63 1
5 9 10 11 14
1
0.89 1
0.74 0.81 1
0.67 0.75 0.76 1
0.45 0.48 0.54 0.73 1
1
0.86 1
0.65 0.75 1
0.64 0.70 0.66 1
0.46 0.48 0.46 0.80 1
1
0.85 1
0.69 0.79 1
0.62 0.72 0.74 1
0.46 0.47 0.54 0.75 1
intron
3' UTR
intergenic

FIGURE S3. The upstream (5') regions of RNA pol. II-bound exons exhibit greater chromatin accessibility. All CDS exons were separated into bins of 1000 exons on the basis of average RNA polymerase II ChIP signal (in stage 5 embryos) across the exon. The location of peak DNaseI accessibility within each exon was identified and the chromatin accessibility within 200 bp of that peak was calculated in 20 bp intervals. Each line in the plot represents the mean chromatin accessibility for 1000 CDS exons colored according to mean RNA pol II ChIP signal for that cohort. The exons with the highest chromatin accessibility were associated with the highest levels of RNA polyermase II ChIP signal, and the highest CDS exon accessibility was associated with even greater accessibility upstream of the exon.

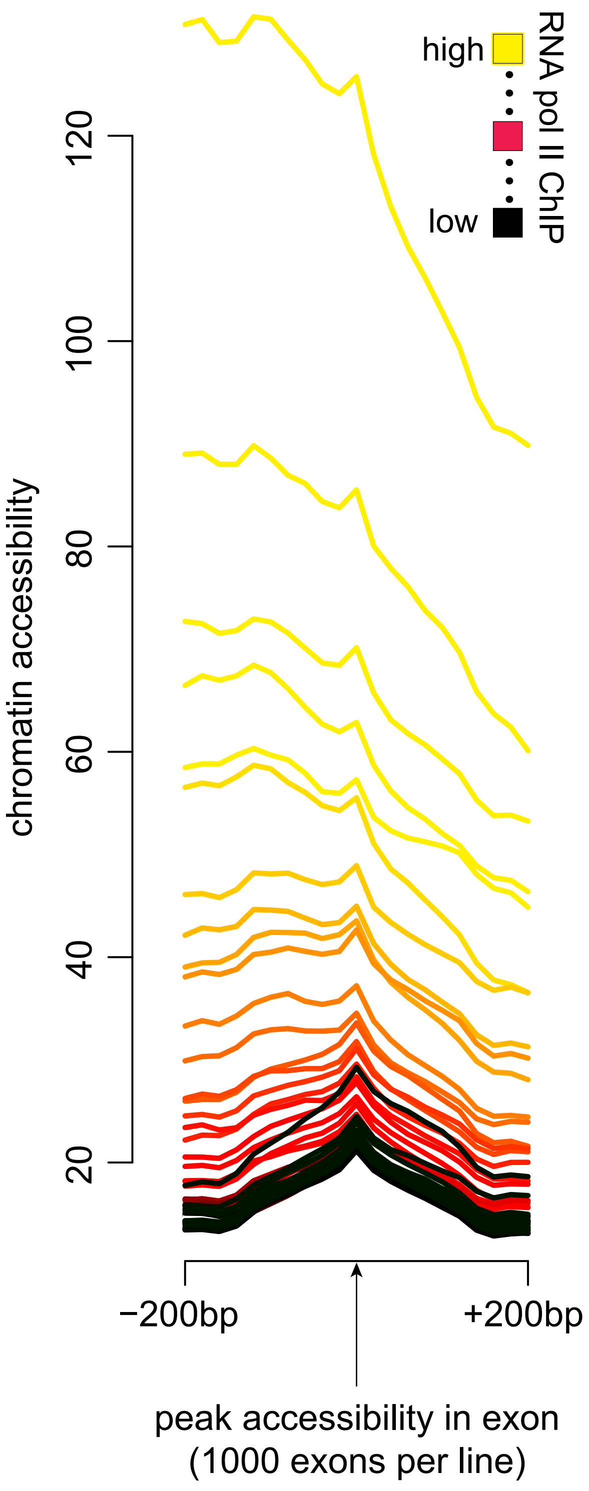
chromatin accessibility
120
100
80
60
40
20
high
low
RNA pol II ChIP
−200bp
+200bp
peak accessibility in exon
(1000 exons per line)

FIGURE S4. Exon expression at 0-2 hours is linked to chromatin accessibility. (a) All exons greater than 320 bp in length with 5' regions free of any other exons within 300 bp were clustered into 4 clusters using kmeans according to peak DNase I cleavage density scores across the 5' exon boundary from low (I) to high (IV). **(b) Chromatin accessibility is associated with mRNA expression over the CDS exon.** For each class of exon, the average expression over each CDS exon between 0-2h of embryonic development was calculated. Then for each class, the % of all genes that were highly-expressed genes was determined (expression signal >=25). Those exons with the highest accessibility had the highest expression between 0-2 hours. ***(c)* Chromatin accessibility is associated with RNA polymerase II binding over the CDS exon.** For CDS exons in each accessibility cluster, the mean levels of RNA polymerase II ChIP signal was calculated over the length of the exon, supporting a correlation between accessibility and active transcription of exons.

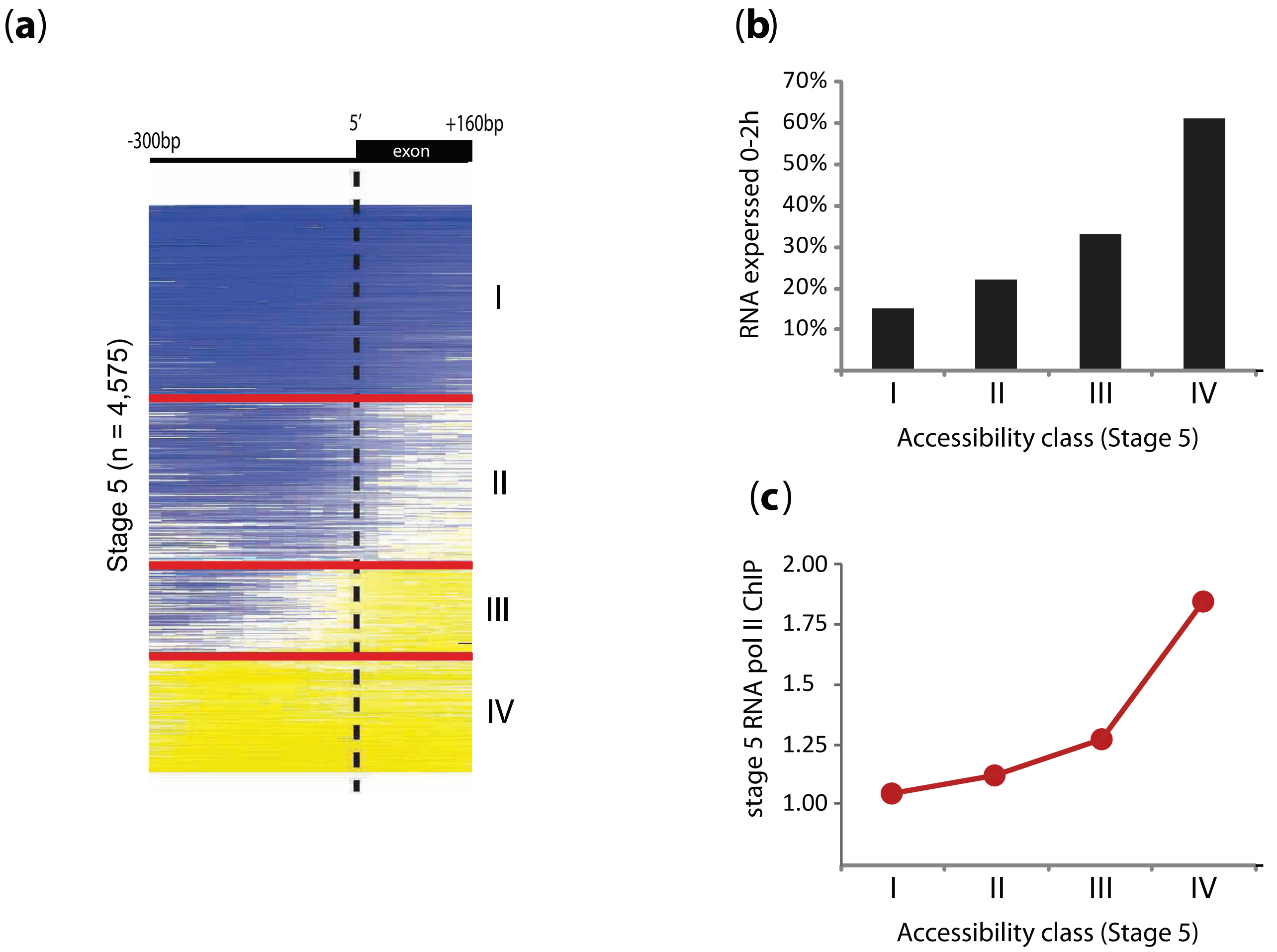
(a)
-300bp
5′
+160bp
exon
Stage 5 (n = 4,575)
I
II
III
IV
(b)
RNA experssed 0-2h
70%
60%
50%
40%
30%
20%
10%
I
II
III
IV
Accessibility class (Stage 5)
(c)
stage 5 RNA pol II ChIP
2.00
1.75
1.5
1.25
1.00
I
II
III
IV
Accessibility class (Stage 5)

FIGURE S5. Developmentally Dynamic Elements cluster into domains with similar patterning. Each DDE was assigned an 'early' or 'late' designation (Table 2) and compared to DDEs separated by variable numbers of DDEs (1 indicates that each DDE was compared to its nearest DDE, 2 indicates that each DDE was compared to the second-nearest DDE). If both DDEs in question were designated similarly it was counted as a success. The y-axis plots the number of successes observed for all comparisons (red line) against the distribution of successes observed using 100,000 draws from a binomial model. The similarity of DDEs separated by up to 5 other DDEs (median distance of 39 kb) was significant, and as distance between DDEs increases the number of successes approached the random model.

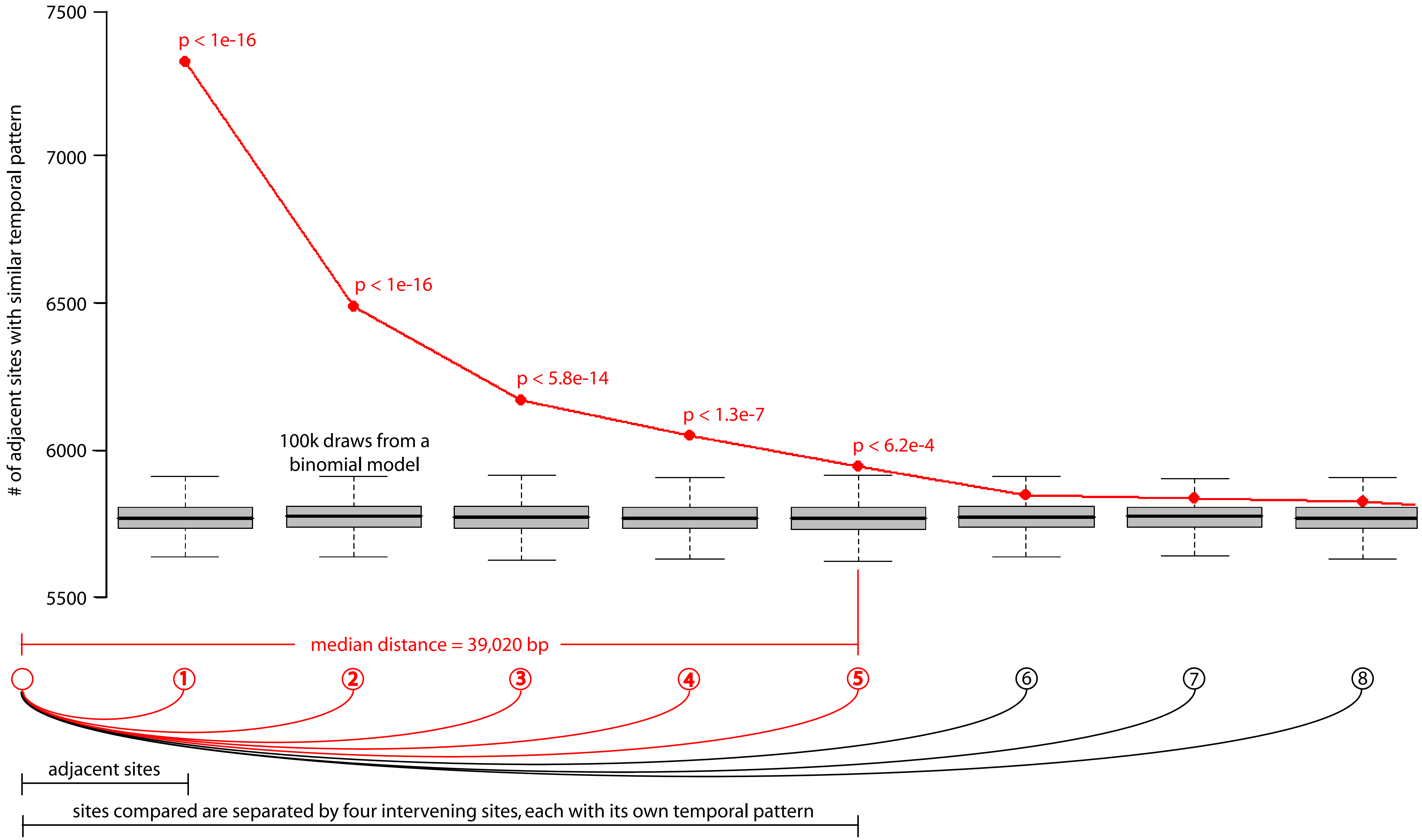

7500
7000
6500
6000
5500
# of adjacent sites with similar temporal pattern
p < 1e-16
p < 1e-16
p < 5.8e-14
p < 1.3e-7
p < 6.2e-4
100k draws from a binomial model
median distance = 39,020 bp
1
2
3
4
5
6
7
8
adjacent sites
sites compared are separated by four intervening sites, each with its own temporal pattern

FIGURE S6. Developmentally Dynamic Domains are enriched in regulatory genes. Genes were ranked (left to right) from highest to lowest DDE density. The height of each red bar indicates the percent of genes within a given 200-gene cohort that was annotated as having a role in transcriptional regulation. Genes with the highest DDE density were enriched in transcription factors.

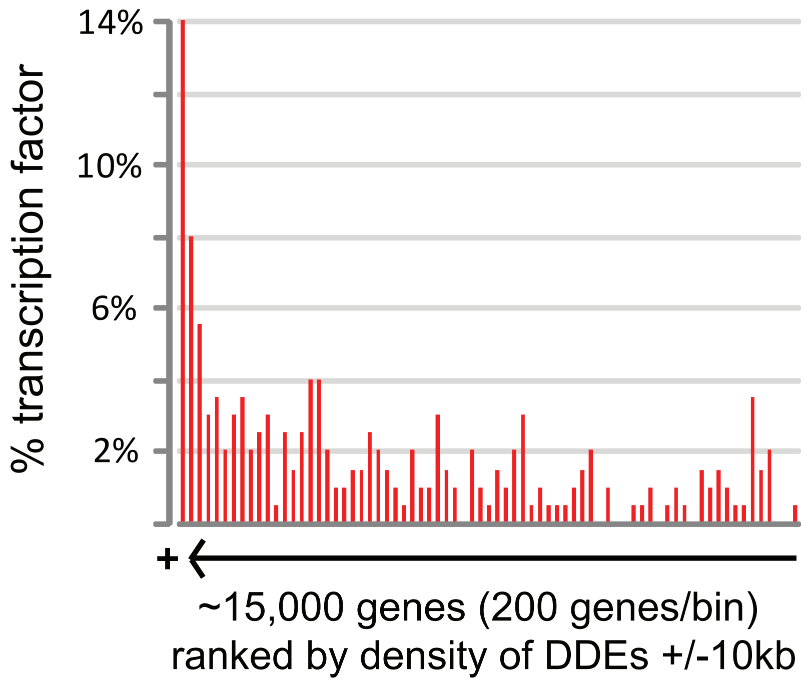
14%
10%
6%
2%
% transcription factor
+
~15,000 genes (200 genes/bin)
ranked by density of DDEs +/-10kb

FIGURE S7. Temporal patterns of chromatin accessibility are associated with the spatiotemporal expression of adjacent genes. BDGP expression annotation terms were derived for the gene whose transcription start site was closest to each DDE in each of the 64 clusters. The mean chromatin accessibility profile for each DDE cluster from Figure 3 is reproduced at the bottom (blue = low, yellow = high), with the mean accessibility of early stages placed above that of later stages. To the right on along the y-axis, all expression terms that were significantly enriched are shown, sorted by developmental stage, with the color of the text for each term indicating the developmental stage at which the term applies (stage 5, 9, 10, 11 and 14 - green, orange, red, blue, purple). The hypergeometric probability associated with a cluster and expression term is indicated by circles whose diameters are proportional to the -log p-values shown in the bottom right. For example, there are more large circles for late embryo tissues above the late accessible clusters than there are for early expression annotation terms (right most columns). The largest circles represent the very conservative Bonferroni-corrected significance ($\alpha=0.01$), while the smallest circles indicate significance on the level of an individual test ($p<0.01$).

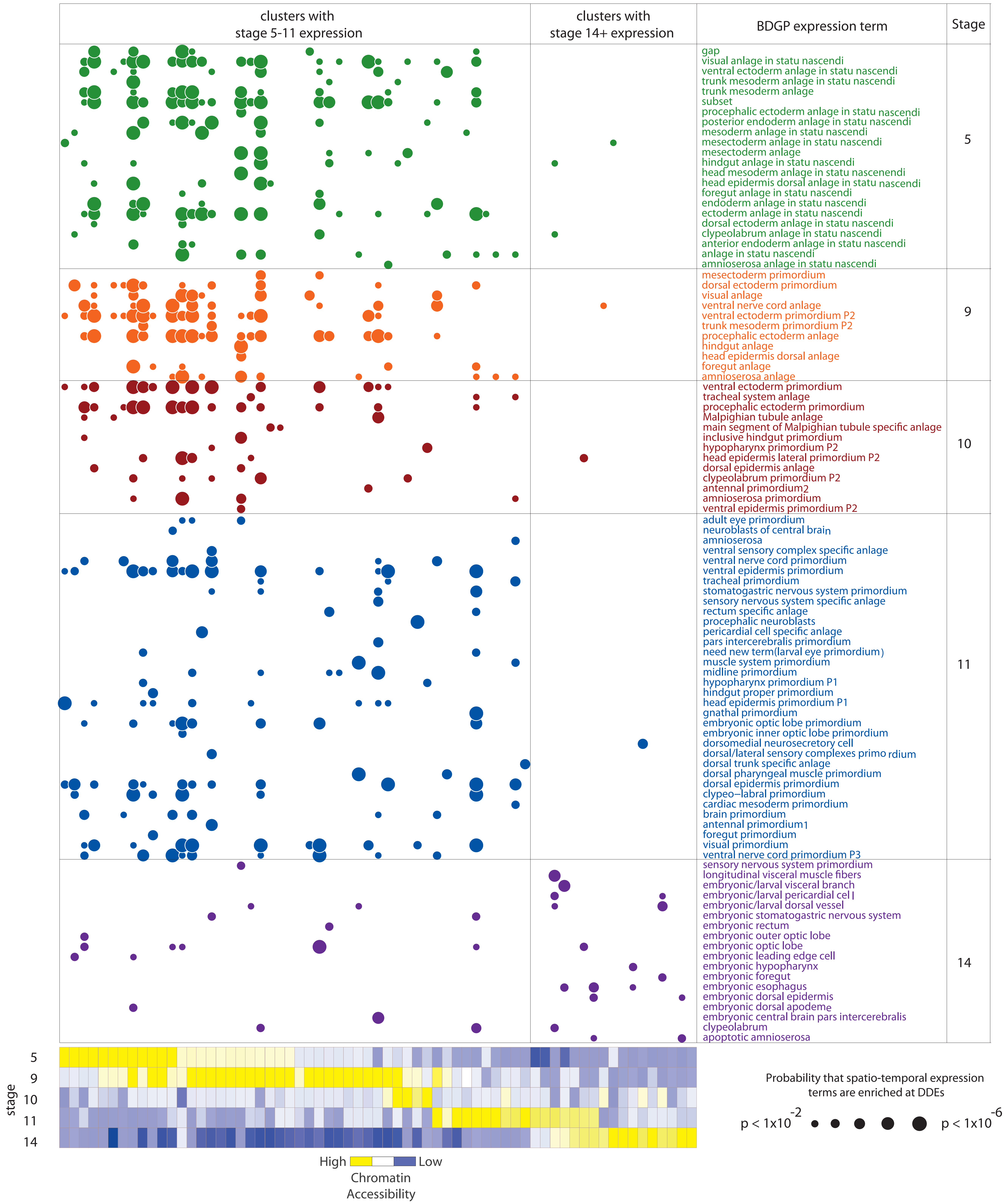
clusters with stage 5-11 expression
clusters with stage 14+ expression
BDGP expression term
Stage
gap
visual anlage in statu nascendi
ventral ectoderm anlage in statu nascendi
trunk mesoderm anlage in statu nascendi
trunk mesoderm anlage
subset
procephalic ectoderm anlage in statu nascendi
posterior endoderm anlage in statu nascendi
mesoderm anlage in statu nascendi
mesectoderm anlage in statu nascendi
mesectoderm anlage
hindgut anlage in statu nascendi
head mesoderm anlage in statu nascenendi
head epidermis dorsal anlage in statu nascendi
foregut anlage in statu nascendi
endoderm anlage in statu nascendi
ectoderm anlage in statu nascendi
dorsal ectoderm anlage in statu nascendi
clypeolabrum anlage in statu nascendi
anterior endoderm anlage in statu nascendi
anlage in statu nascendi
amnioserosa anlage in statu nascendi
5
mesectoderm primordium
dorsal ectoderm primordium
visual anlage
ventral nerve cord anlage
ventral ectoderm primordium P2
trunk mesoderm primordium P2
procephalic ectoderm anlage
hindgut anlage
head epidermis dorsal anlage
foregut anlage
amnioserosa anlage
9
ventral ectoderm primordium
tracheal system anlage
procephalic ectoderm primordium
Malpighian tubule anlage
main segment of Malpighian tubule specific anlage
inclusive hindgut primordium
hypopharynx primordium P2
head epidermis lateral primordium P2
dorsal epidermis anlage
clypeolabrum primordium P2
antennal primordium2
amnioserosa primordium
ventral epidermis primordium P2
10
adult eye primordium
neuroblasts of central brain
amnioserosa
ventral sensory complex specific anlage
ventral nerve cord primordium
ventral epidermis primordium
tracheal primordium
stomatogastric nervous system primordium
sensory nervous system specific anlage
rectum specific anlage
procephalic neuroblasts
pericardial cell specific anlage
pars intercerebralis primordium
need new term(larval eye primordium)
muscle system primordium
midline primordium
hypopharynx primordium P1
hindgut proper primordium
head epidermis primordium P1
gnathal primordium
embryonic optic lobe primordium
embryonic inner optic lobe primordium
dorsomedial neurosecretory cell
dorsal/lateral sensory complexes primordium
dorsal trunk specific anlage
dorsal pharyngeal muscle primordium
dorsal epidermis primordium
clypeo–labral primordium
cardiac mesoderm primordium
brain primordium
antennal primordium1
foregut primordium
visual primordium
ventral nerve cord primordium P3
11
sensory nervous system primordium
longitudinal visceral muscle fibers
embryonic/larval visceral branch
embryonic/larval pericardial cell
embryonic/larval dorsal vessel
embryonic stomatogastric nervous system
embryonic rectum
embryonic outer optic lobe
embryonic optic lobe
embryonic leading edge cell
embryonic hypopharynx
embryonic foregut
embryonic esophagus
embryonic dorsal epidermis
embryonic dorsal apodeme
embryonic central brain pars intercerebralis
clypeolabrum
apoptotic amnioserosa
14
stage
5
9
10
11
14
High
Low
Chromatin Accessibility
Probability that spatio-temporal expression terms are enriched at DDEs
p < 1x10-2
p < 1x10-6
